# Supplementary material for: Wnt‐dependent spatiotemporal reprogramming of bone marrow niches drives fibrosis
Source: Hemasphere. 2026 Feb 20;10(2):e70309. doi: 10.1002/hem3.70309 (PMC12921528; doi:10.1002/hem3.70309)
Supplement: Supplementary file 1 — Supporting Information [file HEM3-10-e70309-s002.docx]

**Supplemental Materials and Methods**

**Wnt-dependent spatiotemporal reprogramming of**

**bone marrow niches drives fibrosis**

Bella Banjanin^#^, James Nagai^#^, YeVin Mun^#^, Stijn Fuchs, Inge Snoeren, Joachim Boers, Mayra L. Ruiz Tejada Segura, Hector Tejeda Mora, Anna Katharina Galyga, Adam Benabid, Rita Sarkis, Olaia Naveiras, Marta Rizk, Michael Wolf, Rogerio B. Craveiro, Fabian Peisker, Ursula Stalmann, Jessica E. Pritchard, Hosuk Ryou, Nasullah Khalid Alham, Marek Weiler, Fabian Kiessling, Twan Lammers, Anna Rita Migliaccio, Kishor Kumar Sivaraj, Ralf H. Adams, Eric Bindels, Joost Gribnau, Daniel Royston, Hélène F.E Gleitz^*^, Rafael Kramann^*^, César Nombela-Arrieta^*^, Ivan G. Costa^*^, Rebekka K. Schneider^*$^

# contributed equally

* contributed equally

**$Correspondence**

reschneider@ukaachen.de

**List of Supplementary Materials**

#### Supplemental Materials and Methods

Fig S1 to S8

**Fig S1 The combination of stromal Cre drivers provides high granularity of the bone marrow niche**

**Fig S2 CAR cells acquire a pro-fibrotic phenotype but are reduced in frequency while fibroblasts expand in bone marrow fibrosis**

**Fig S3 Distinct phenotype switch in TPO-OE induced bone marrow fibrosis in the diaphyseal and metaphyseal localization**

**Fig S4 OLC as progenitor cells of the bone marrow are enriched in the metaphysis in bone marrow fibrosis**

**Fig S5 Stromal stem and progenitor cells are skewed in their differentiation in bone marrow fibrosis**

**Fig S6 NCAM is a marker for fibrosis-driving cells in bone marrow fibrosis**

**Fig S7 Wnt signaling is upregulated in metaphyseal stromal progenitor cells and CAR cells**

**Fig S8 Inhibition of Wnt signaling reduces fibrotic transformation and osteoclerosis in BM fibrosis**

**Tables**

**Table S1** List of differentially expressed (DE) genes between TPO-OE and EV, related to figure 2

**Table S2** Gene sets related to Figures 5 and 8

**Table S3** Human cohort characteristics related to Figure 6

#### **Supplemental Materials and Methods**

#### **Single-Cell Proportion Analysis**

Differential Proportion Analysis was performed using the package scProportion (Version 0.0.0.9; https://github.com/rpolicastro/scProportionTest), accessing the cell abundance difference in the phenotypes presented in the study.

#### **scRNAseq niche localization prediction with cell deconvolution and label transfer**

We made use of laser capture data (LCM-seq) composed of five distinct bone marrow niches in homeostasis (Arteries, SubEndosteum, Endosteum, High sinusoids, and Low sinusoids) to recover the spatial context from the scRNA-seq data (1), samples from sinusoid sections were disregarded due to low mapping of signatures as the sinusoidal bulkRNAseq contained mainly hematopoietic cells. We defined a gene expression signature for each niche by using the ROC statistics from Seurat. We considered all genes presenting a ROC value lower than 0.3 or larger than 0.7. We then used these signatures as reference for deconvolution analysis by providing single-cell data from Homeostasis samples as input with a CIBERSORTx algorithm (2) implementation provided in (<https://github.com/veltenlab/rnamagnet>,(1)). We summed up the probabilities for all cells in a cluster, providing a niche score for each cell type. For recovery of metaphyseal and diaphyseal location signatures, we made use of TransferLabels in Seurat, using a published scRNA-seq dataset containing both metaphyseal and diaphyseal BM stromal populations (3).

#### **Osteogenesis, Chondrogenesis and Adipogenesis genesets**

The osteo/adipo genesets were obtained from Supplemental Figure S7 (4) and chondro geneset from Supplementary Table S2 (5). The median of the genesets gene expression was compared using the Wilcoxon Test. We have added these datasets in Table S1.

#### **Coefficient of variation (CV) scoring - SPEC (differentiated) and MONO (progenitor) scores**

We explored the coefficient of variation (CV) score to measure the stem-ness or specialization state of a cell. For every gene, the CV is calculated as the standard deviation divided by average expression. Low CV is indicative of genes with low variance (i.e. not being cell specific), while high CV indicates cell specific genes (as cells start expressing specific genes once they decide on a fate). We next perform a z-score transformation of the gene CV scores. Genes with z-score below 0.5 are defined as low CV genes, while genes with z-score > 0.5 as high CV genes. Only genes expressed in more than 50 cells are considered. We finally obtain a CV-low score per cell as the total expression value of low-CV genes, and the CV-high score the total expression of high-CV genes per cell. High-CV scores (SPEC score) indicate the level of specialization of the cell, i.e. cells whose gene expression is dominated by cell specific genes. Low-CV scores indicate cells with monotonous (or stem-like properties), as they express genes common to most cells (MONO score).

####

#### **Reconstructing cell development trajectories**

For trajectory analysis, we used phateR implemented in the Seurat package (runPHATE) with k=20 and the four Phate components (6). Next, the Phate components were used to re-cluster the cells, and the trajectories were defined using the new clusters and the CV score described above. As described in the ArchR package, the trajectories were obtained by computing the smooth::spline with the N-dimensional coordinates and the pseudo-time values.

#### **Ligand-receptor (LR) analysis**

For ligand-receptor analysis, we used the CellPhoneDB method and a LR consensus database implemented in the LIANApy package (Version 0.1.10,(7)). We performed the analysis per phenotype (Homeo, EV, and TPO). We only consider statistically significant LR pairs (p-value < 0.01). We next used CrossTalkeR(Version 1.3.2,(8)) to characterize major phenotype-specific cell-cell interactions. We only considered cell pairs, in which the number of LR pairs changes significantly (Fisher’s Test, p-value < 0.05) between two compared conditions. We used GO Biological Process 2011 database (available in https://maayanlab.cloud/Enrichr/#libraries) to subset pathway-specific (Wnt) LR interactions.

#### **Ligand-receptor (LR) category-based filtering**

Cell communication scores obtained through CrossTalkeR (8) were subset according to the Ligand classification from the Matrissome database (9) or the Cytosig database (9), and the following cell niches:

Metaphysis = ["OLC1", "OLC2", "OLC3", "Pre_chondro", "Chondro", "FB1", "FB2", "FB3", "FB5", "FB6", "FB7", "OB"]

Transition = ["Epi", "FB4", "IFN_FB", "CAR7_ribo", "Mus_CAR", "FB8", "Stroma", "Endo2", "Mural", "Endo1", "Smooth_Mus"]

Diaphysis = ["IFN_CAR", "CAR1", "CAR2", "CAR4_Pdg", "CAR3", "CAR6", "CAR5"]

Hematopoietic (Hemap) = [“Hemap_prog”, “Neutro1”, “Neutro2”].

The significance of the differences between the distributions of subset cell communication scores in different conditions (TPO and EV) was addressed through two-sided Wilcoxon tests as implemented in the Scipy library (version 1.9.1) in Python(10).

#### **Bone marrow computational pathology quantification MarrowQuant**

The MarrowQuant (mouse version) algorithm was employed for bone marrow tissue quantifications. Within QuPath 0.3.2, annotations were performed following the methods described in previous studies (11, 12). In brief, tissue boundaries encompassed most of the tissue, excluding highly hemorrhagic regions. The bone area was excluded from the analysis, and the total marrow area is defined as the selected region of interest minus the bone and artifact areas therefore defined as the denominator. The MarrowQuant algorithm was subsequently executed, with a minimum size threshold set at 120µm2 for adipocytes. The resulting quantification data were exported in a .txt file. Codes and tutorials are available on the following GitHub link: https://github.com/orgs/Naveiras-Lab/repositories

#### **StarDist on Adipocytes**

For the individual segmentation of bone marrow adipocytes and tracking of their size distribution, we utilized the StarDist model trained specifically for bone marrow adipocytes, as detailed in Sarkis et al. 2023 (11). This algorithm was also integrated within QuPath, and the same tissue boundaries defined for MarrowQuant quantifications above were applied to StarDist. Consequently, StarDist accurately detected the individual adipocytes. Subsequently, a classification algorithm was employed, and the quantification results were exported using a .txt file. Codes and tutorials are available on the following GitHub link: https://github.com/orgs/Naveiras-Lab/repositories.

#### **microCT scans of tibia and analysis**

A Bruker Skyscan 1272 microCT scanner equipped with a 11 megapixel sensor was used. The following parameters were chosen for scanning: 4x4 µm^2^ pixel size and a matrix of 1344x2016. The voltage was 60 kV and the current 166 µA. Furthermore, a 0.25 mm Al filter was applied. The exposure time per image was set to 1558 ms and 8 averages per projection were acquired. The step between projections was set to 0.2° and a total of 180° was imaged, resulting in 900 projections per scan. Data reconstruction and beam hardening corrections (up to 70%) were performed using the NRecon software (Bruker, Belgium). Morphometric and densitometric analysis were done in CTan software (Bruker, Belgium) inside a cylindrical VOI (7.07 mm^2^ x 1.6 mm) located in the upper part of tibia from the section where secondary spongiosa forms a sole uninterrupted region. A sequence of image processing steps was applied to virtually separate the trabeculae area from cortical bone. After bone thresholding, the outer surface of the tibia was defined by the ROI-shrink wrap method. Inside this ROI, the inverted binarized image was treated by a morphological escalation method (opening and closing with a stepwise increasing parameter from 2 up to 10px). After removal of residual speckles, the region of trabeculae was defined. Then, 3D morphological analyses were performed on the new binarized image inside this trabeculae VOI and following parameters were obtained: bone volume to total volume (BV/TV); trabecular number (Tr.N); trabecular thickness (Tr.Th) and connectivity of trabeculae (Connectivity).

**Quantification of tdTomato cells**

All image stacks were processed using FIJI (13). Briefly, images (n=3 per condition) were contrast enhanced, normalized, and converted to 8-bit grayscale. Contrast enhancement and normalization were performed to standardize the intensity levels across all images, ensuring comparability. The intensity profile along multiple lines perpendicular to the bone surface was measured to ensure representative sampling across the bone surface. All profile line measurements were done from the start of the growth plate border, which corresponded to the highest recorded intensity values. The total integrated density along the distance for each condition was calculated by averaging the integrated density values of all lines across all images. The area under the curve (AUC) for each condition was calculated using the trapezoidal rule after the growth plate border. Statistical significance between conditions was determined using a t-test, with a p-value <0.05 considered significant.

#### **Quantification of NCAM1/CD56-stained IHC murine sections**

All images were analyzed using ImageJ2 version 2.14.0. Briefly, three representative regions of the BM per sample were selected using the rectangle tool, and total tissue area was measured after color thresholding. Afterwards, we made use of the ‘color deconvolution’ plugin for ‘FastRed/FastBlue/DAB’ to retrieve the signal from the DAB channel (NCAM1/CD56). The threshold for the positive signal of DAB was determined using automatic thresholding schemes per image, and measured as the percentage area of the image.

#### **Visiopharm image analysis of NCAM1/CD56-stained human sections**

The Visiopharm Integrator System (VIS) platform version 2023.01 was used to analyze digitized serial human BM slides. Image analysis protocols are implemented as Analysis Protocol Packages (APP) in VIS. Several APPs were designed to quantify slides stained with CD56. Prior to the image analysis, it was important to outline the Region of Interest (ROI), hence a number of auxiliary APPs were designed to detect ROIs. To detect ROIs, the first auxiliary APP ran on the slide using threshold classification to identify the tissue regions. The second auxiliary APP ran on the slide using the DeepLabv3 network of the VIS AI module to identify the bone and peeled regions of the biopsy. For the analysis, an APP ran on 20X on the slides using the U-Net network of the VIS AI module to identify NCAM1/CD56-positive staining. As a post-processing step, a threshold of 22 pixels away from bone or peeled areas was used to classify the detected NCAM1/CD56 positive staining into two classes and output variables obtained from the APPs include: total tissue area, total NCAM1/CD56 positive area, NCAM1/CD56 positive area close to bone or peeled areas and NCAM1/CD56 positive area away to bone or peeled areas. The CIF score was determined as previously described (14).

#### **Prospective sorting of NCAM1+ and VCAM1+ BM stromal cells**

For the prospective sorting of NCAM1/CD56+ and VCAM1/CD106+ BMSCs, we processed samples as above in ‘Isolation of BM stromal cells for scRNA sequencing’, and sorted lineage/CD31 negative alive singlets NCAM1+ or VCAM1+. After three passages the cells were immortalized using Large-T virus and characterized using flow cytometry and RT-qPCR.

#### **NCAM1 and VCAM1 stromal cell co-culture with cKit cells**

c-Kit+ hematopoietic stem and progenitor cells were isolated from WT mice using the autoMACS pro Separator after crushing the compact bone (Miltenyi Biotec). c-Kit+ cells were seeded on top of immortalized NCAM1+ or VCAM1+ cBMSCs in StemSpan™ Serum-Free Expansion Medium (Stem Cell Technology, Vancouver, Canada) supplemented with murine thrombopoietin (m-tpo) (50 ng/mL; Peprotech), murine stem cell factor (m-scf) (50 ng/mL; Peprotech). After 24 hours of co-culture, the non-adherent hematopoietic cells were collected for apoptosis flow cytometry (Annexin V APC Apoptosis Detection Kit, eBioscience). All samples were analyzed by flow cytometry using a FACS Fortessa (BD Biosciences, San Jose, CA). Data were analyzed using FlowJo software (Version 10, TreeStar Inc.)

**RNA extraction and real-time qPCR analysis**

RNA from cBMSCs was extracted using Trizol solution (ThermoFisher) according to the manufacturer’s instructions, and 1,0 μg of total RNA was reverse transcribed using the high-capacity cDNA Reverse Transcription kit (Applied Biosystems). Quantitative polymerase chain reactions were performed with SYBRGreen PCR master mix (ThermoFisher) on an Biorad CFX384 Real-Time PCR System. Glyceraldehyde-3-phosphate dehydrogenase (Gapdh) was used as a housekeeping gene. Data was analyzed using the -Δct method. Primers are listed in the table below.

**Table 1.** RT-qPCR primers

| **Name** | **Forward Primer** | **Reverse Primer** |
| --- | --- | --- |
| Gapdh | AGGTCGGTGTGAACGGATTTG | TGTAGACCATGTAGTTGAGGTCA |
| Dcn | TTCCTACTCGGCTGTGAGTC | AAGTTGAATGGCAGAACGC |
| CD34 | AGCGGTACAGGAGAATGCAG | ATCAGGCAGAGAGCTACCCA |
| Ly6a | CCCCTACCCTGATGGAGTCT | AGAGGTCTTCCTGGCAACAG |
| Cxcl12 | CACTCCAAACTGTGCCCTTCA | CACTTTAATTTCGGGTCAATGC |
| Lpl | ATGGATGGACGGTAACGGGAATGT | TGGATAATGTTGCTGGGCCCGATA |
| Vcam1 | CCGGCATATACGAGTGTGAA | GATGCGCAGTAGAGTGCAAG |
| Kitl | TTATGTTACCCCCTGTTGCAG | CTGCCCTTGTAAGACTTGACTG |
| Cd200 | CCATCTCTCCACCTACAGCC | TGCAGCGCCTTTCTTTCATC |

**Visium spatial transcriptomics sample preparation**

Visium CytAssist Spatial Gene expression assay (10X Genomics, PN #1000523) was conducted following 10X Genomics recommendations. Briefly, three FFPE sections were placed onto each capture area of a 11mm2 Visium CytAssist slide (three experimental groups per slide, three replicates per experimental group). Following section placement, deparaffinization, H&E staining, brightfield imaging, decrosslinking and library preparation were performed. Libraries were sequenced on a NovaSeq sequencer (Illumina) using sequencing depth and parameters recommended by 10X Genomics.

**Visium spatial transcriptomics analysis**

For each bone presented in each slide, a segmentation file was generated as recommended by 10x Genomics using Loupe Browser. Next, sequencing reads and the alignment files were processed using spaceranger v.3.0.0. Seurat was used to filter low quality cells (nFeature_Spatial>200 & nFeature_Spatial < 7500 & percent.mt < 5) per sample. Data integration was done using harmony and spatial domains (SpDs) were identified using the FindCluster with resolution=0.7.

**Single cell RNA sequencing of Pyrvinium Tosylate (PT) treatment cohort**

The scRNA-seq count matrix was obtained by aligning the raw sequencing reads into the mm10 mouse reference genome via the cellranger (version 8.0.1). Seurat (version 5.0.1) was used to filter low quality cells (nFeature_Spatial>200 & nFeature_Spatial < 7500 & percent.mt < 5) per sample. Data integration was done using harmony and single cell clusters were identified using the FindCluster with leiden clustering using resolution=0.1. Stroma cells’ clusters were further sub-clustered using FindCluster with leiden clustering using resolution=0.2.

**Figure S1 (related to figure 1): The combination of stromal Cre drivers provides high granularity of the bone marrow niche**

(A) FACS plots showing tdTomato expression in the different fractions of the bone marrow (BM) per Cre-driver. EV= empty vector control; TPO=Thrombopoietin-induced bone marrow fibrosis

(B) Schematic of anatomical structures and niches in the bone marrow

(C) Whole dataset before removal of skeletal cells, hematopoietic cells

(D) Expression of tdTomato transcript per Cre-reporter

(E) Dot plot of top 5 markers per cluster of re-clustered stromal dataset


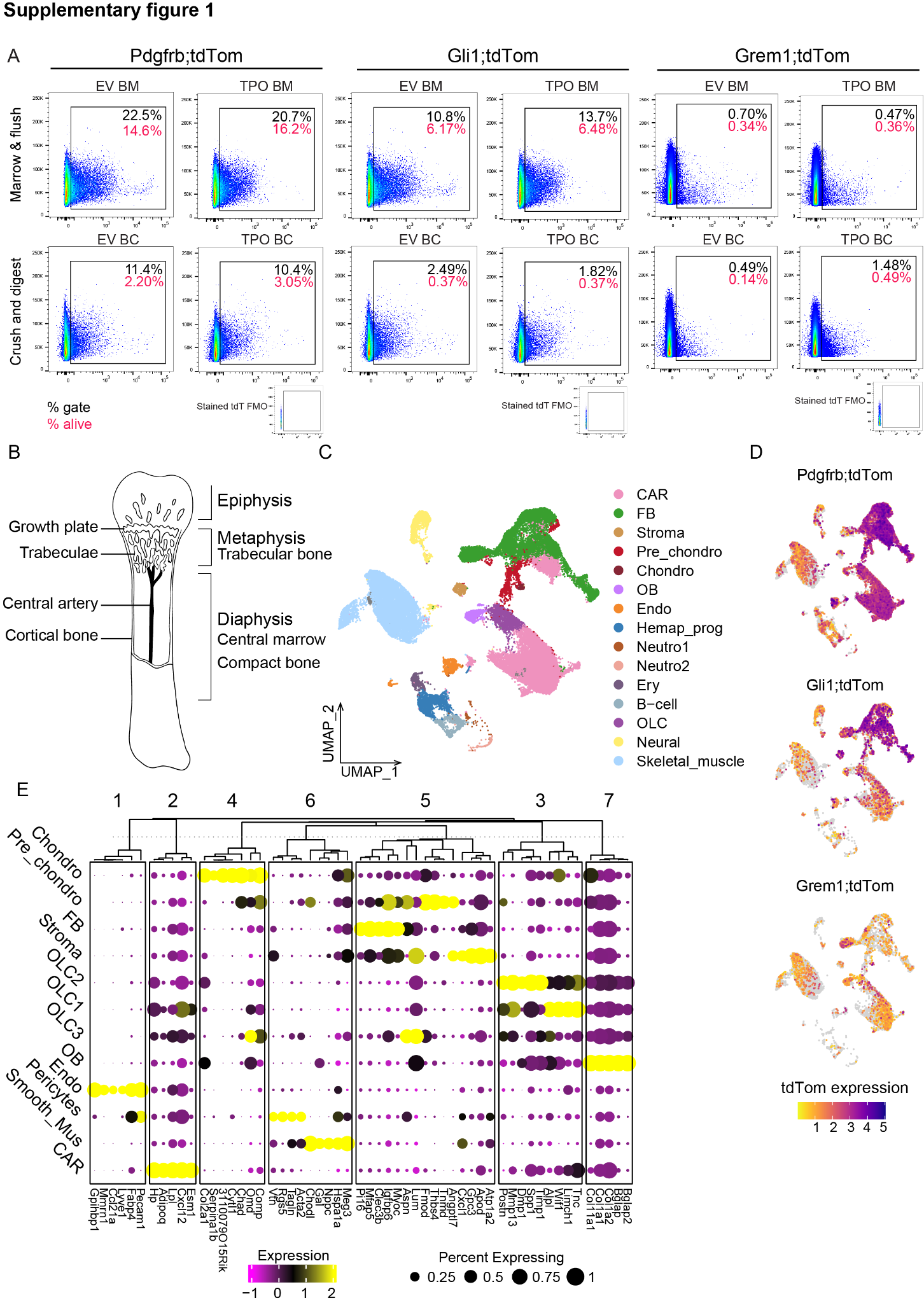


**Figure S2 (related to Figure 2): CAR cells acquire a pro-fibrotic phenotype but are reduced in frequency while fibroblasts expand in bone marrow fibrosis**

For panels A, B, C and D: Gli1;tdTom: EV n=5, TPO-OE n = 6, Grem1;tdTom: EV n=4 TPO-OE n=4, Pdgfrb;tdTom: EV n=3, TPO-OE n=4.

(A) Blood counts of experimental mice used for 10X scRNA sequencing, t-test performed per genotype group ( Pdgfrb;tdTom, Gli1;tdTom, Grem1;tdTom). (B) Cell counts of the bone marrow (BM cellularity) at the end of the experiment of 10X cohort; t-test performed per genotype group ( Pdgfrb;tdTom, Gli1;tdTom, Grem1;tdTom).

(C) Spleen weight/body weight at the end of the experiment of 10X cohort. t-test performed per genotype group ( Pdgfrb;tdTom, Gli1;tdTom, Grem1;tdTom.

(D) Fibrosis grading (reticulin grade) of BM of 10X cohort. t-test performed per genotype group ( Pdgfrb;tdTom, Gli1;tdTom, Grem1;tdTom).

(E) Proportion changes in Homeo versus EV obtained using scProportionTest.

(F) Violin Plot of Ly6a expression per cluster per condition

(G) Donor chimerism after transplant, n=5 mice per group in the Ly6a;GFP recipient transplant; In brief, Ly6a;eGFP mice were lethally irradiated and intravenously received c-kit-enriched HSCs from WT littermates expressing either thrombopoietin cDNA (TPO-OE; n = 5, three males) or control cDNA [empty vector, EV, n = 5; both lentiviral SFFV-iblue fluorescent protein (BFP) vector backbone] as outlined in Figure 2A.

(H) Hematological phenotype in Ly6a-eGFP transplanted animals comparing to the empty vector; (EV) control condition (grey) to Thrombopoietin-overexpression (TPO-OE; purple); n=5 mice per group.

(I) BM cell counts (bone marrow cellularity) and spleen size expressed as spleen to body weight ratio. One-way ANOVA with multiple comparisons.

(J) Stromal cells isolated from bone chips of Ly6a-GFP mice show strong GFP signal in culture as indicated by flow cytometry.

(K) Representative images of EV control and TPO-OE BM of Ly6a;GFP mouse, Endomucin in red, DAPI nuclear stain in blue, scale bar = 100µm

Statistical significance is indicated by: *p<0.05, **p<0.01, ***p<0.001, ****p<0.0001.


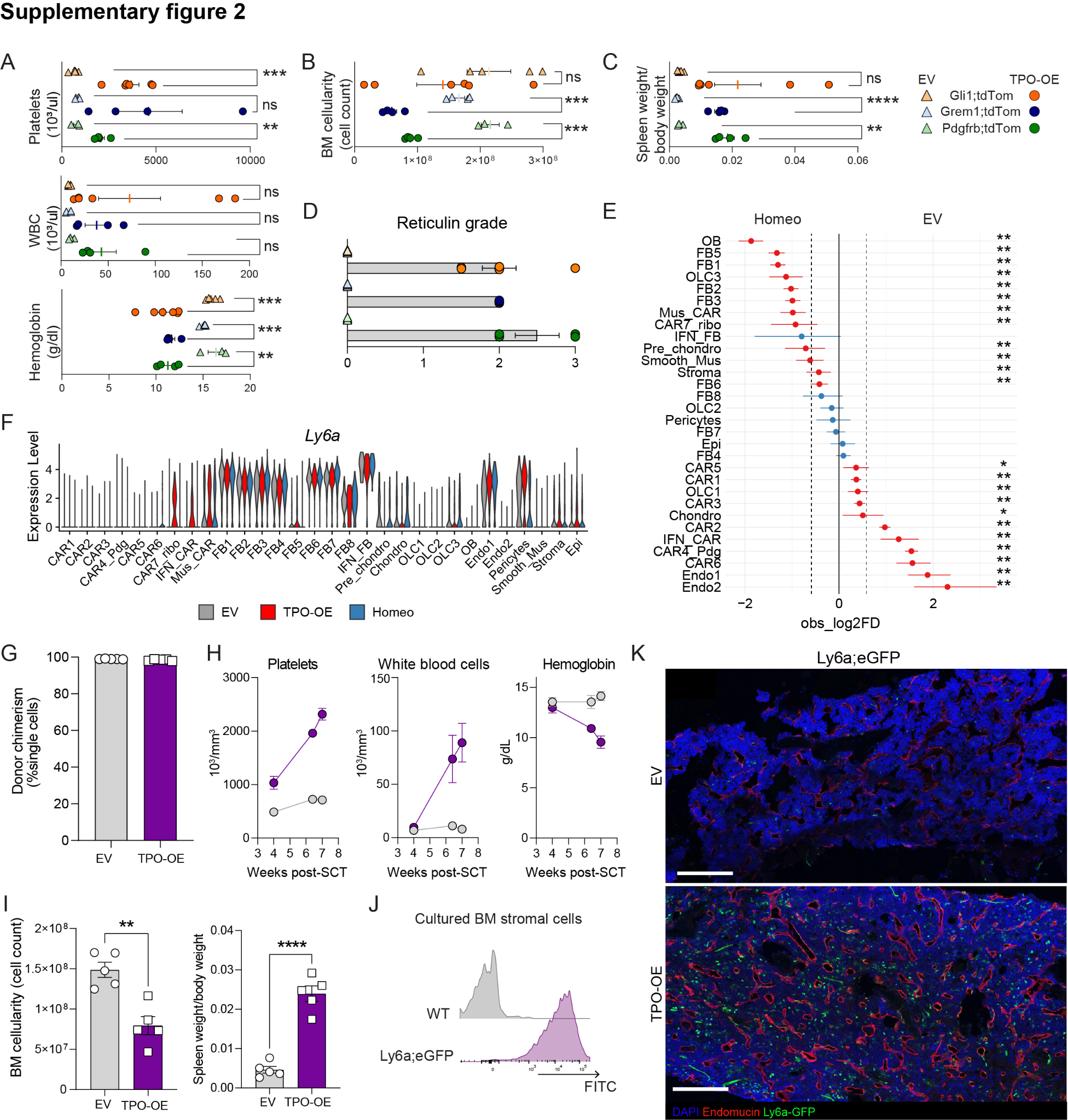


**Figure S3 (related to Figure 3) Distinct phenotype switch in TPO-OE induced bone marrow fibrosis in the perivascular (diaphysis) and endosteal (metaphysis) localization**

1. Zoom in of peri-trabecular region in TPO-OE (fibrosis) transplanted Pdgfrb;tdTom reporter mice showing region of osteosclerosis (new bone formation), marked with podoplanin staining and typical morphology of osteo-lineage cells/osteoblasts. Scale bar: 50µm.
2. Whole-mount imaging of Grem1;tdTom in the control (EV) and fibrosis (TPO-OE) condition. Reticular-like cells emerging from growth plate (GP) indicated with yellow arrow head. Dotted line = growth plate. Scale bar: 50µm
3. Volcano plot showing differentially expressed genes (TPO vs EV) in the endothelial cluster endo-1. Apelin (Apln) is highlighted in green.
4. Gene set enrichment of differentially expressed genes in endo-1.
5. Confocal images of fibrotic bigenic PdgfrbCreER;tdTomato depict the peritrabecular region as a hotspot zone in bone marrow fibrosis in particular in comparison to the central marrow (diaphyseal) region (zoom in). Demounted and reticulin stained whole mount bone (shown in E). The hotspot tdTomato-positive areas overlap with increased reticulin deposition (peritrabecular region/metaphysis), Representative regions of interest (ROIs) are shown along the tibia. Scale bar: 500µm.
6. Grading of reticulin fibrosis grade according to WHO criteria in the metaphysis (meta) and diaphysis (dia) in femurs of mice transplanted with ckit-enriched cells transduced with the MPLW515L mutation.
7. Quantification of Gli1;tdTom+ cell expansion/movement away from growth plate (GP) border in femurs of mice transplanted with ckit-enriched cells transduced with the MPLW515L mutation. Statistical test: two-sample t-test on area under curve (auc), with a p-value <0.05 considered significant.


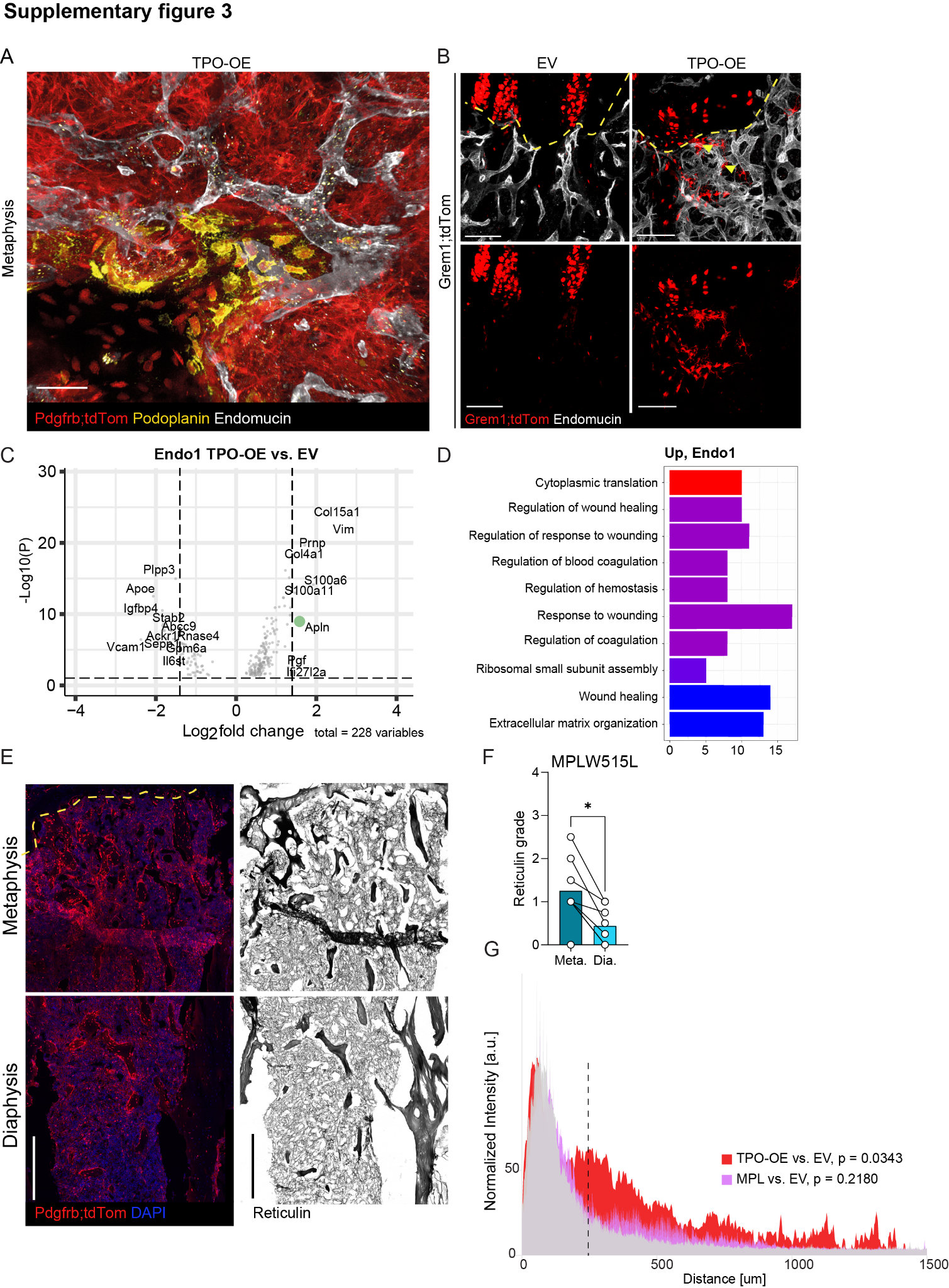


**Figure S4 (related to Figure 4) Stromal differentiation trajectories**

(A) PHATE dimension reduction differentiation trajectory highlighting the distribution of the included clusters as highlighted in the lower panel.

(B) Violin plots of the progenitor (MONO) and differentiated cell (SPEC) score per celltype. X-axis is ordered according to the mean values.

(C) G1 cell cycle analysis of stromal cell clusters, comparing TPO-OE vs. EV control. Statistical comparison was done using Fisher Exact Test.

(D) Progenitor (MONO) and differentiation (SPEC) scores along trajectories defined in Fig. 4C

(E, F) Flow cytometry analysis of TdTomato expression in metaphyseal versus diaphyseal regions in Pdgfrb;tdTom (E) and Gli1;tdTom (F) homeostatic bones, in the right panel quantification of tdTom+ cells per region, paired t-test. M. = metaphysis, D. = diaphysis. Insert shows fluorescence minus one (FMO).

(G) Representative confocal images of aged Pdgfrb;tdTom and Gli1;tdTom mice, 1 year after tamoxifen induction. Note the wide-spread abundance of Pdgfrb;tdTom-lineage cells (diaphysis, metaphyseal region), whereas Gli1;tdTom-lineage cells are located at the metaphyseal region and growth plate. Dotted yellow line represents growth plate (GP)

(H) Proportion test Gli1;tdTom versus Pdgfrb;tdTom, on homeostasis subsetted data, obtained using scProportion test. Relative differences in cell proportion per cluster, red colored dots show significant fold change (FDR<0.05 and absolute fold change > 0.58) with error bars showing confidence intervals for the magnitude difference (permutation test, n=1000). Blue dots- ns; red dots: significant changes.

Statistical significance is indicated by: *p<0.05, **p<0.01, ***p<0.001, ****p<0.0001

**
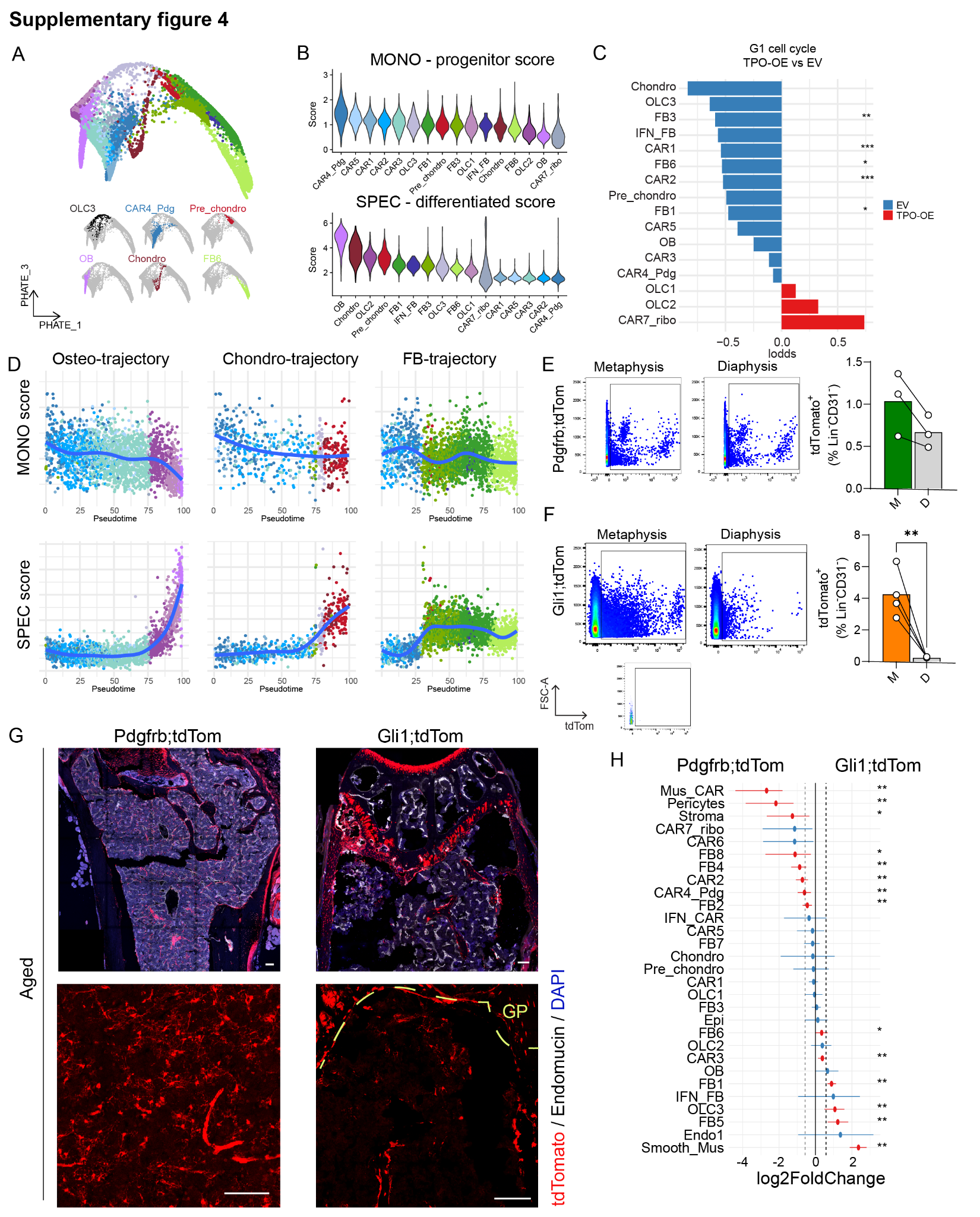
**

**Figure S5 (related to Figure 5): Stromal stem and progenitor cells are skewed in their differentiation in bone marrow fibrosis**

1. “Marrowquant” tissue composition quantification and number of adipocytes in H&E stained WT, JAK2V617F and MPLW515L femurs. One way ANOVA tested.
2. Aggregate expression of chondro-genes per cluster, TPO vs EV, two-sided Wilcox test
3. Heatmap of cluster averages of adipogenesis (adipo), osteogenesis (osteo) and chondro-genes genes of homeostasis subsetted dataset

Statistical significance is indicated by: *p<0.05, **p<0.01, ***p<0.001, ****p<0.0001

**
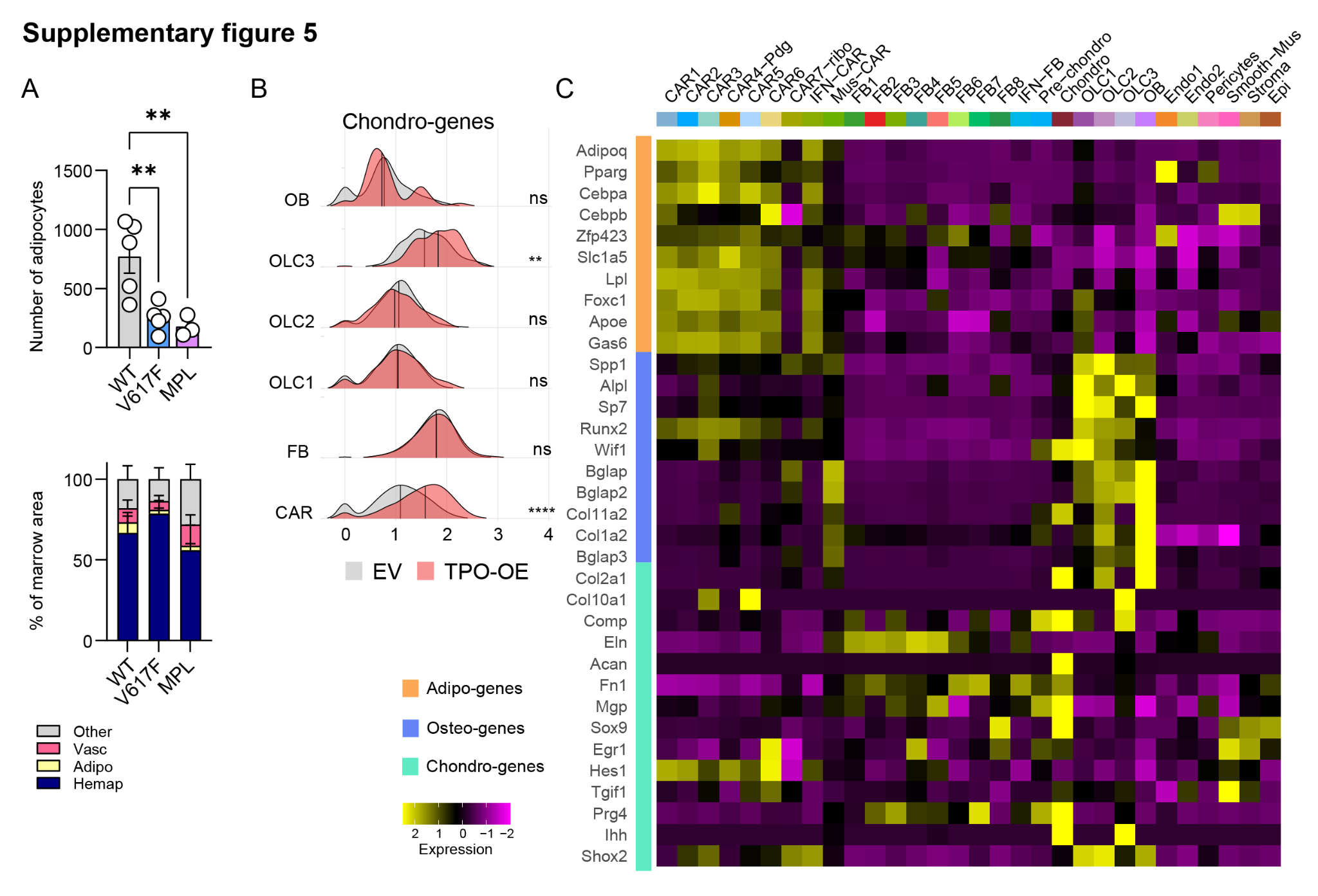
**

**Figure S6 (related to Figure 6): Identification of markers for peritrabecular progenitor cells**

1. Ridge plot of Fibulin1 (Fbln1) expression for clusters
2. Confocal images of TPO-OE transplanted tibias in Gli1;tdTom lineage traced mice, at early harvest (5W post transplantation; post-tx) and at intermediate harvest (8W post-Tx) time point. Composite image shown, the right panels show individual channels of Fbln1 and Gli1;tdTom respectively. Scale bar: 50µm.
3. Ridge plot of Vcam1 expression for clusters.
4. Representative flow cytometry plot showing NCAM1 and VCAM1 expression of lineage depleted BM and digested BC fractions of metaphyseal and diaphyseal fractions of humeri from homeostatic mice (n=3).
5. Quantification of NCAM1+ and VCAM1+ cells found in metaphyseal (meta) and respective diaphyseal (dia) regions of humeri, n=3. Paired t-test. ns:non-significant.
6. Brightfield microscopy of prospectively sort-purified NCAM1+ and VCAM1+ cells respectively, following immortalization, scale bar = 230µm.
7. Percentage of protein marker expression in prospectively sorted NCAM1+ and VCAM1+ cells after culture and immortalization. Proteins as indicated.
8. Heatmap of delta CT of genes of interest shown of prospectively sorted NCAM1+ and VCAM1+ cells after culture and immortalization, at time-point of FACS analysis in panel G.
9. Gating strategy of apoptosis assay (Annexin V combined with DAPI) of CD45+ cells following 24-hour co-culture with NCAM1+ cell line (CL) or VCAM1+ CL.
10. Viability of hematopoietic stem and progenitor cells (cKit-enriched cells) after 24-hour culturing with prospectively sorted NCAM1 or VCAM1 cells, compared to conventional cKit suspension culture. One-way ANOVA.

Statistical significance is indicated by: *p<0.05, **p<0.01, ***p<0.001, ****p<0.0001


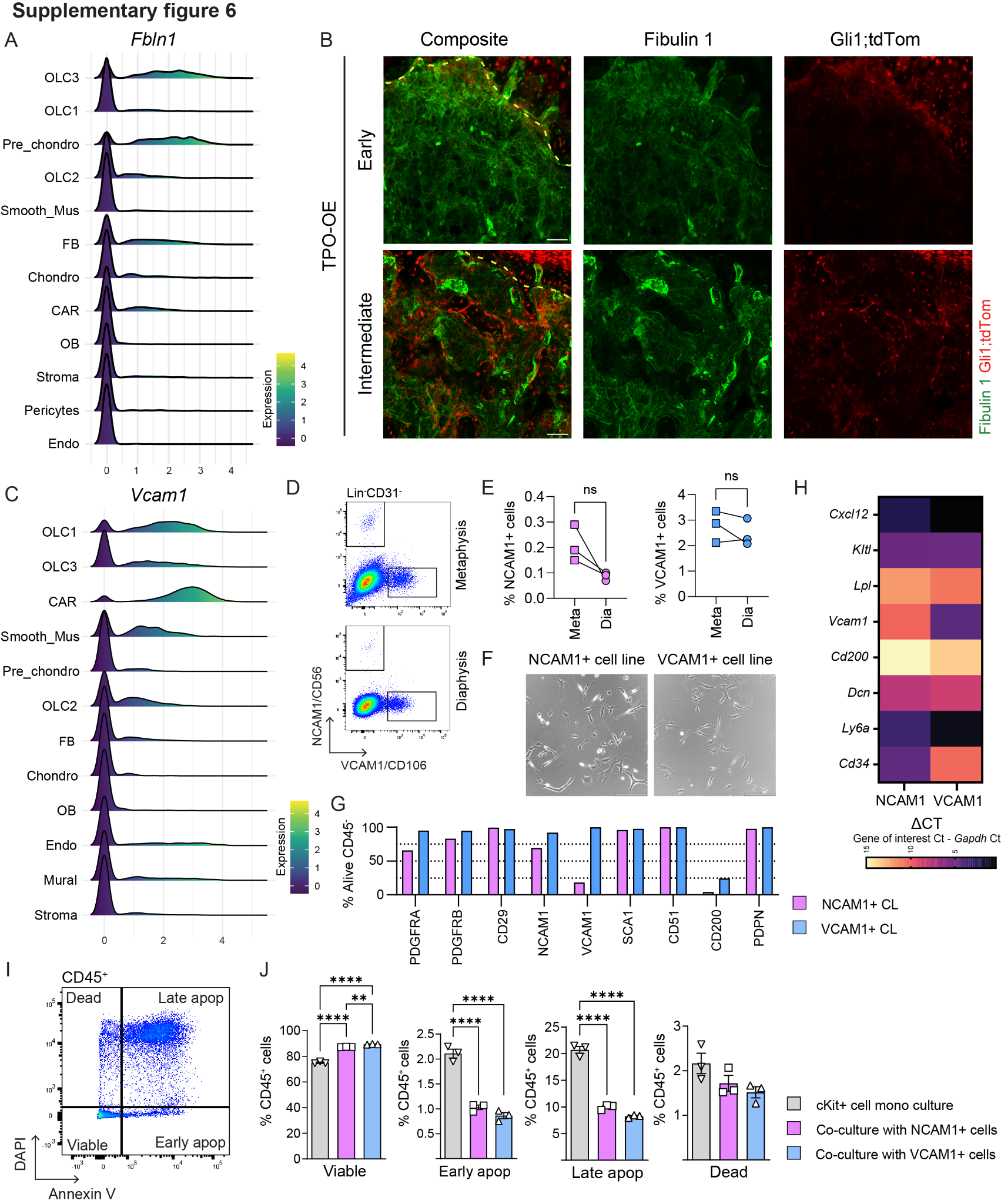


**Figure S7 (related to Figure 7): Wnt signaling is reactivated in peritrabecular mesenchymal progenitor cells**

(A-C) CCI plot signaling showing receptor interactions of the different niches (metaphysis, diaphysis, transition vs. hematopoietic=hemap) but also cell clusters (TPO-OE vs. EV) filtered on genes belonging to chemokines based on CytoSig database (A) ECM regulators based on Matrissome DB (B) and Wnt signaling based on GO (C)

(D) CCI plots of major niches identified in Figure 7, TPO-OE vs. EV

(E) Beta-catenin staining of murine humeri, EV= empty vector, control, MPLW515L = fibrotic marrow. Composite image shown, and the right panels show individual channels of beta-Catenin and Gli1;tdTom respectively. White arrowheads highlight beta-catenin and Gli1;tdTom co-expression.Scale bar = 50µm

(F) Whole tissue quantification of active beta-catenin signal, shown per MF grade, One-way ANOVA.

(G) Grading table for stromal beta catenin grading (sbCG) of human bone marrow biopsies. One-way ANOVA with Kruskal-Wallis. H/R = healthy/reactive, MF = myelofibrosis grade 0-3 Statistical significance is indicated by: *p<0.05, **p<0.01, ***p<0.001, ****p<0.0001

**
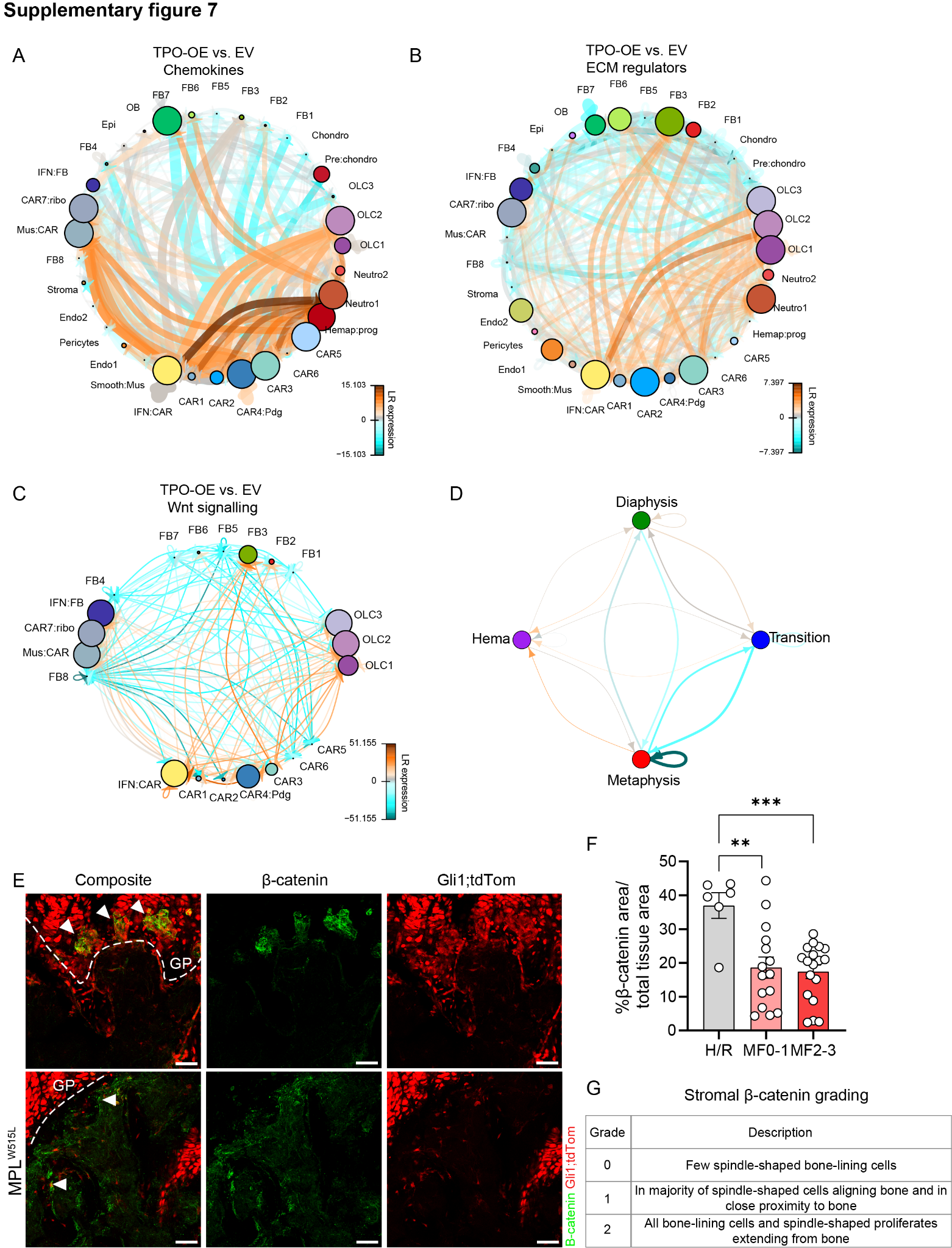
**

**Figure S8 (related to Figure 8): Wnt signaling is reactivated in peritrabecular mesenchymal progenitor cells**

(A) Schematic of experimental design, following BM transplant of cKit-enriched cells transduced with EV-eGFP or TPO-OE-eGFP viral vector, mice were treated twice weekly with Pyrvinium-Tosylate (PT)-DMSO solution or PBS-DMSO as a control starting 21 days after transplantation.

(B) BM chimerism of recipient mice at harvest time-points, three independent cohorts shown

(C) Progression of MPN blood phenotype of cohort in F7E, PLT = platelets, WBC = white blood cells, HGB = hemoglobin. One-way ANOVA performed on last time-point, only significant results shown.

(D) BM cellularity per group, and spleen weight/ body weight. One-way ANOVA performed.

(E) Representative images of reticulin and H&E staining of paraffin-embedded bones in EV control mice

(F) Spleen over body weight at harvest of experimental groups. One-way ANOVA performed.

(G) Representative images of H&E images of spleens, scale bar: 500µm

(H) HSC FACS of cohorts, individual mice per cohorts shown. One-way ANOVA performed. Non-significant differences.

(I) Proportion of cell clusters from TPO-OE_PBS and TPO-OE_PT dataset (from figure 8C) associated with tdTom-lineage cell clusters (from figure 1B)

(J) Remainder of violin plots of figure 8D, median depicted with circle, wilcox test for significance.

(K) Violin plot of Pf4 expression in SpDs. MK (megakaryocyte); Fib (fibroblast); CAR-cells; OLC (osteolineage cells); IFN (interferon); endo (endothelium)

(L) Ridge plots of remaining SpDs of figure 8J, K, L. TPO-OE PBS vs EV PBS in the left panel, TPO-OE PT vs TPO-OE PT in the right panel. One sided Wilcox test performed per spatial domain cluster, per comparison.

Statistical significance is indicated by: *p<0.05, **p<0.01, ***p<0.001, ****p<0.0001


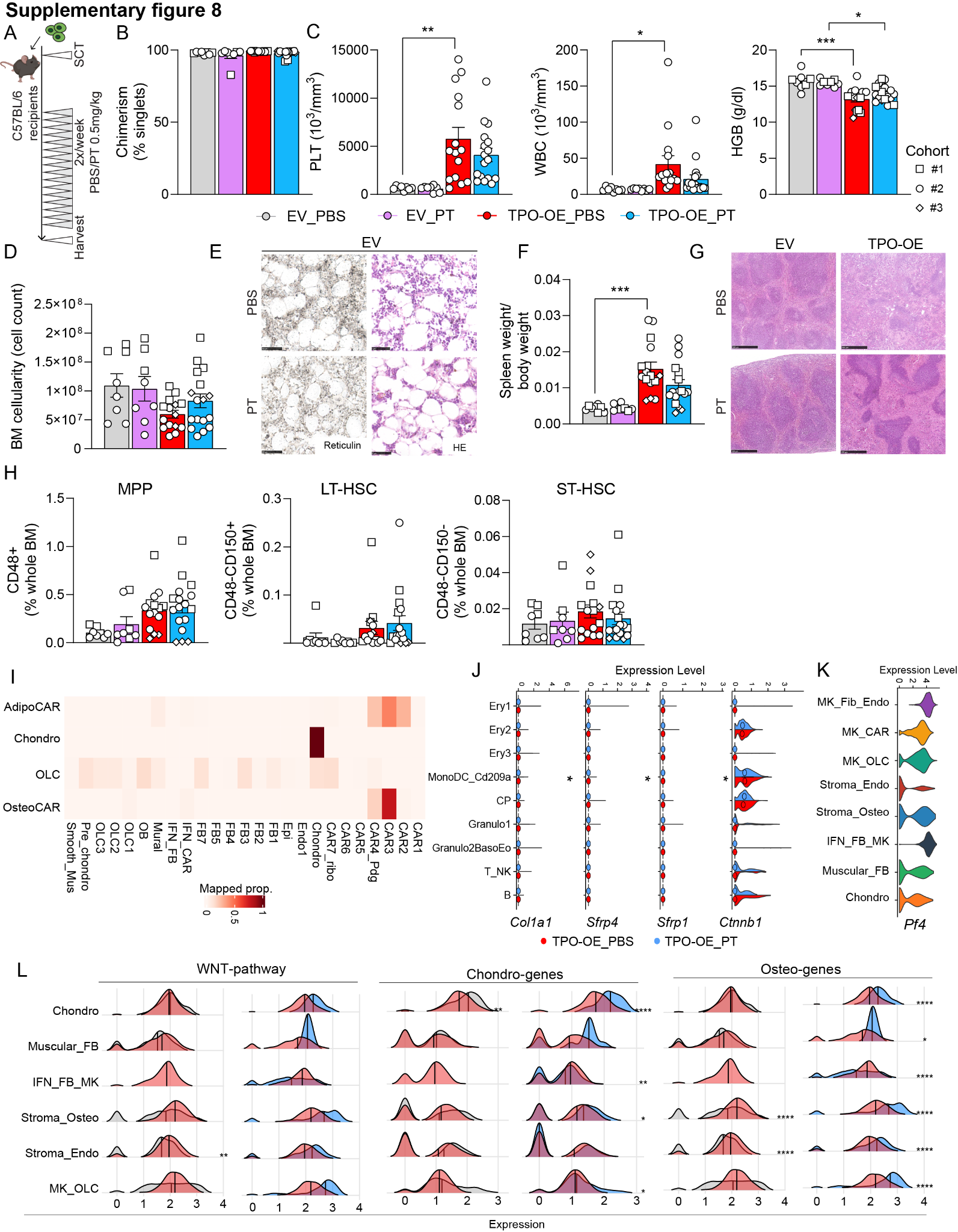


**References**

1. [Baccin C, Al-Sabah J, Velten L, Helbling PM, Grünschläger F, Hernández-Malmierca P, et al. Combined single-cell and spatial transcriptomics reveal the molecular, cellular and spatial bone marrow niche organization. Nat Cell Biol. 2020 Jan;22(1):38–48.](http://paperpile.com/b/CGmOud/s6fhJ)

2. [Newman AM, Steen CB, Liu CL, Gentles AJ, Chaudhuri AA, Scherer F, et al. Determining cell type abundance and expression from bulk tissues with digital cytometry. Nat Biotechnol. 2019 May 6;37(7):773–82.](http://paperpile.com/b/CGmOud/sf7A7)

3. [Sivaraj KK, Majev PG, Jeong HW, Dharmalingam B, Zeuschner D, Schröder S, et al. Mesenchymal stromal cell-derived septoclasts resorb cartilage during developmental ossification and fracture healing. Nat Commun. 2022 Jan 28;13(1):571.](http://paperpile.com/b/CGmOud/Eagz1)

4. [Sivaraj KK, Jeong HW, Dharmalingam B, Zeuschner D, Adams S, Potente M, et al. Regional specialization and fate specification of bone stromal cells in skeletal development. Cell Rep. 2021 Jul 13;36(2):109352.](http://paperpile.com/b/CGmOud/H1sod)

5. [Coates BA, McKenzie JA, Buettmann EG, Liu X, Gontarz PM, Zhang B, et al. Transcriptional profiling of intramembranous and endochondral ossification after fracture in mice. Bone. 2019 Oct;127:577–91.](http://paperpile.com/b/CGmOud/QJlP8)

6. [Moon KR, van Dijk D, Wang Z, Gigante S, Burkhardt DB, Chen WS, et al. Visualizing structure and transitions in high-dimensional biological data. Nat Biotechnol. 2019 Dec;37(12):1482–92.](http://paperpile.com/b/CGmOud/310tV)

7. [Dimitrov D, Türei D, Garrido-Rodriguez M, Burmedi PL, Nagai JS, Boys C, et al. Comparison of methods and resources for cell-cell communication inference from single-cell RNA-Seq data. Nat Commun. 2022 Jun 9;13(1):1–13.](http://paperpile.com/b/CGmOud/l3sLX)

8. [Nagai JS, Leimkühler NB, Schaub MT, Schneider RK, Costa IG. CrossTalkeR: analysis and visualization of ligand-receptorne tworks. Bioinformatics. 2021 Nov 18;37(22):4263–5.](http://paperpile.com/b/CGmOud/1VONH)

9. [Jiang P, Zhang Y, Ru B, Yang Y, Vu T, Paul R, et al. Systematic investigation of cytokine signaling activity at the tissue and single-cell levels. Nat Methods. 2021 Sep 30;18(10):1181–91.](http://paperpile.com/b/CGmOud/Kt5bb)

10. [Virtanen P, Gommers R, Oliphant TE, Haberland M, Reddy T, Cournapeau D, et al. SciPy 1.0: fundamental algorithms for scientific computing in Python. Nat Methods. 2020 Feb 3;17(3):261–72.](http://paperpile.com/b/CGmOud/goEeu)

11. [Sarkis R, Burri O, Royer-Chardon C, Schyrr F, Blum S, Costanza M, et al. MarrowQuant 2.0: A Digital Pathology Workflow Assisting Bone Marrow Evaluation in Experimental and Clinical Hematology. Mod Pathol [Internet]. 2023 Apr [cited 2023 Oct 2];36(4). Available from:](http://paperpile.com/b/CGmOud/p3yFI) <https://pubmed.ncbi.nlm.nih.gov/36788087/>

12. [Tratwal J, Bekri D, Boussema C, Sarkis R, Kunz N, Koliqi T, et al. Across Aging and Aplasia: A Digital Pathology Workflow for Quantification of Bone Marrow Compartments in Histological Sections. Front Endocrinol . 2020 Sep 24;11:480.](http://paperpile.com/b/CGmOud/BEqf9)

13. [Schindelin J, Arganda-Carreras I, Frise E, Kaynig V, Longair M, Pietzsch T, et al. Fiji: an open-source platform for biological-image analysis. Nat Methods. 2012 Jun 28;9(7):676–82.](http://paperpile.com/b/CGmOud/jmCP)

14. [Ryou H, Sirinukunwattana K, Aberdeen A, Stolz BJ, Byrne H, Harrington HA, et al. Continuous Indexing of Fibrosis (CIF): improving the assessment and classification of MPN patients. Leukemia. 2022 Dec 5;37(2):348–58.](http://paperpile.com/b/CGmOud/9CtZ)
